# Posture Estimation Using Surface Electromyography during Wheelchair Hand-Rim Operations

**DOI:** 10.3390/s22093296

**Published:** 2022-04-25

**Authors:** Satoshi Ohashi, Akira Shionoya, Keiu Harada, Masahito Nagamori, Hisashi Uchiyama

**Affiliations:** 1 Information and Management Systems Engineering, Nagaoka University of Technology, 1603-1 Kamitomioka, Nagaoka 940-2188, Niigata, Japan; shionoya@vos.nagaokaut.ac.jp (A.S.); nagamori@vos.nagaokaut.ac.jp (M.N.); utiyama@vos.nagaokaut.ac.jp (H.U.); 2 Department Information Science and Engineering, National Institute of Technology, Tomakomai College, 443 Aza-Nishikioka, Tomakomai 059-1275, Hokkaido, Japan; keiu@tomakomai-ct.ac.jp

**Keywords:** parasports, assistive technology, competitive wheelchair, sEMG, iEMG, muscle activation, seat pressure

## Abstract

This study examined competitive wheelchairs that facilitate sports participation. They can be moved straight ahead using only one arm. Our designed and developed competitive wheel-chairs have a dual hand-rim system. Their two hand-rims, attached to a drive wheel on one side, can be operated simultaneously for straight-ahead movement. Specifically, based on integrated electromyography (iEMG) data calculated from surface electromyography (sEMG), we examined the wheelchair loading characteristics, posture estimation, and effects on body posture during one-arm propulsion movement. The first experiment yielded insights into arm and shoulder-joint muscle activation from iEMG results obtained for two-hand propulsion and dual hand-rim system propulsion. Results suggest that muscle activation of one arm can produce equal propulsive force to that produced by two arms. The second experiment estimated the movement posture from iEMG during one-arm wheelchair propulsion. The external oblique abdominis is particularly important for one-arm wheelchair propulsion. The iEMG posture estimation validity was verified based on changes in the user body axis and seat pressure distribution. In conclusion, as confirmed by iEMG, which is useful to estimate posture during movement, one-arm wheelchair use requires different muscle activation sites and posture than when using two arms.

## 1. Introduction

As audiences across the world tuned in to watch the Paralympic Games, they saw athletes using impressive para-sport equipment such as high-technology wheelchairs, prosthetic limbs, and other assistive technology [1,2,3,4]. For sports played by people with disabilities, research and development of sports equipment according to the events, the sites and degrees of disability, physique, and other conditions are accelerating worldwide [5,6,7,8,9,10,11]. Although the characteristics of such research and development are rarely disclosed to the public, the results are expected to be developed eventually into commercial products [12,13].

The research and development for competitive wheelchairs has also considered lighter and stronger materials such as aluminum, titanium, and carbon [14]. Differences in frame materials and hand-rim shape alone have led to great differences in mechanical workload and exercise physiology [15,16,17]. Furthermore, competition-specific sports wheelchairs must be configured and adapted in numerous ways to suit an athlete’s physical impairments and to improve performance and comfort. Physical disability types include limb deficiency, impaired muscle power, impaired range of movement, ataxia, and athetosis. Wheelchair hand-rim operation requires arm and hand muscle function. Individuals with disabilities in one arm or hand exhibit markedly lower performance than users of two arms [18]. Conventionally used manual wheelchairs require long and intensive periods of use and control of two arms until proper hand-rim operation can be achieved. Therefore, some adaptation is necessary for users with asymmetrical arm use. Moreover, propelling a wheelchair with one arm during competition is an even more difficult task when pushing a hand-rim with two hands. Some such individuals with disabilities often require one-arm drive wheelchairs [19,20]. Nevertheless, only a few reports of the relevant literature describe studies related to one-arm driven wheelchairs; even these reports have contents related to driving systems or persons with hemiplegic disabilities [21,22,23,24,25,26]. No research report describes such a system for competitive wheelchairs.

This study was conducted to examine a competitive wheelchair for practical application, operable by moving straight ahead with only one arm, which will allow participation in sports. The wheelchair with a dual hand-rim system we developed has two hand-rims attached to the drive wheel on one side [27,28]. A user moves in a straight line by gripping both hand-rims simultaneously with one hand. Alternatively, a user can turn using a single hand-rim. The wheelchair presents the added benefit that it can be operated with one arm, even for movements during competition, to execute sudden stops and starts: stop-and-go motion. Such maneuverability enables one-arm movements such as going forwards, backwards, turning, and stopping, which can be done using propulsion with two arms. The results can support many possibilities: for example, if a competitor who still has function of two arms uses this wheelchair, then the wheelchair could be used to drive straight ahead using the same sports motion as that of an athlete holding a racket in one hand while operating the dual hand-rim; also, athletes who can move only one arm can operate the wheelchair just as one might operate a regular dual-arm wheelchair. Sports motions of this kind were not possible with conventional hand-rim operation. The benefits obtained from this study can support and improve athletes’ operations and competitive skills.

Despite the benefits explained above, no report of the relevant literature has described a study of competitive wheelchairs equipped with dual hand-rim systems. Consequently, many unanswered questions remain about loads on the body during hand-rim operation and the loads’ effects on user posture. Once these issues are resolved, it will clarify the conditions of compatibility with the competition and will also allow for training according to the site and degree of disability. Our competitive wheelchair with dual hand-rim system will have a different hand-rim operation than existing competition wheelchairs. Therefore, as a preliminary step in the investigation under subdivided conditions, the first step is necessary to clarify the differences in general muscle activation and movement posture by comparing one-arm with two-arm during the simplest straight-ahead movement. This is because differences in seat height and axle position are known to affect propulsion efficiency, stability, and wheelchair manageability [29], and estimation of biomechanical parameters during straight-line driving and evaluation of the operability of a competitive wheelchair are important issues for users with disabilities [30]. An earlier study used findings from surface electromyography (sEMG) of users to characterize and elucidate wheelchair propulsion, because a correlation exists between sEMG data and muscle strength [31]. The strength of the primary muscles in the user’s upper limb musculature strongly influence the propulsive force transmitted to the wheelchair [32]. In the case of one-arm operation, there should be a difference in muscle activation between the left and right sides. In other words, this difference in muscle activation is related to changes in the motion of the upper limb. As described herein, we thought that by identifying the site of muscle activation by sEMG it would be possible to estimate approximate movement posture. This knowledge is important for the development of new competitive wheelchairs and their use in sports. Many methods have been proposed for detecting motion posture, including two-dimensional and three-dimensional video analyses based on computer vision and motion capture technology [33,34]. However, our methods do not require great resources of equipment, cost, or time, in addition to burdensome preparation for experiments.

As described herein, we present an estimate of movement posture during one-arm operation of a wheelchair based on changes in integrated electromyography (iEMG) data calculated from sEMG data. The first experiment is designed to elicit insights into differences in muscle activation of a user’s arm and shoulder joint muscles when using one arm and when using two arms with competitive wheelchair equipped with a dual hand-rim system. The experimentally obtained muscle activation results suggest that one arm use can produce equal propulsive force to that produced using two arms. The second experiment was conducted using iEMG data to estimate the movement posture during wheelchair propulsion with one arm. The external oblique abdominis play an important role in producing the movement posture for wheelchair propulsion force with one arm. Finally, using iEMG data, the posture estimation was verified by assessing the amount of change in the user’s body axis and seat pressure distribution. Results demonstrate that one-arm operation uses different muscle activation sites than the body posture used for propulsion by two-arm, indicating the body posture differences estimated from surface EMG.

## 2. Materials and Methods

This study was conducted to develop competitive wheelchairs that will allow participating in sports by facilitating straight-ahead movement with one arm. For example, our wheelchair is intended for use in situations where the athlete grips a racket in one hand and operates the hand-rim with the other hand, or when the athlete must propel the competitive wheelchair with only one arm. However, the differences in muscle activation and body posture between one-arm and two-arm operations have not been clarified. Once these issues are resolved, it will be possible to determine the muscles needed to strengthen one-arm operation and the approximate range of adaptability to the site and degree of disability. Preliminary test results confirmed great differences in body postures during straight-line motion with one-arm propulsion. In the case of one-arm operation, there should be a difference in muscle activation between the left and right sides. In addition, this difference in muscle activation is related to changes in the motion of the upper limb. We thought that by identifying the site of muscle activation by sEMG, it would be possible to estimate approximate movement posture. The first experiment was conducted to elicit insights into differences in muscle site activity from iEMG results obtained for one-arm and two-arm propulsion of a wheelchair equipped with a dual hand-rim system. Based on results of the first experiment, the second experiment tested whether body posture can be estimated from iEMG data during one-arm wheelchair propulsion based on the results of seat pressure distribution and changes in body axis.

### 2.1. Competitive Wheelchair with a Dual Hand-Rim System

**Figure 1:**
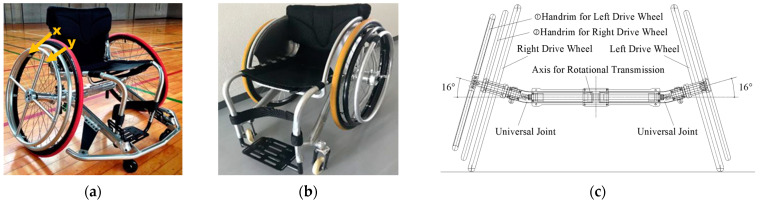
Competitive wheelchair with the dual hand-rim system: (**a**) right-hand drive with a camber angle, (**b**) left-hand drive without a camber angle, and (**c**) a double-ring drive shaft structure.

Figure 1 shows a competitive wheelchair with a dual hand-rim system designed for our study. Such wheelchairs are driven by a double-ring drive shaft structure [27,28]. Two wheelchairs are used for this research: (a) one with a right-hand drive with a camber angle and (b) one with a left-hand drive without a camber angle. These wheelchairs are de-signed to be interchangeable between the right and left sides by reassembly of parts. Figure 1c shows a driving force transmission axle (DFTA) and universal joint that were developed to transmit the driving force from the operation of the outer hand-rims to the oppo-site drive wheel in a competitive wheelchair with a camber angle. The material used for the DFTA is standard internal iron with specific gravity of 7.87 g/cm^3^ and Young’s modulus of 192.08 GP. A steel universal joint of the same standard is attached to the DFTA on each side. This universal joint has a structure in which the rotational transmission speed is not constant with the rotation angle, but which repeats the speed increase and decrease in a 180-degree rotation cycle. Therefore, by installing two universal joints with rotational phases that are 90 degrees apart, the rotational speed to the opposite drive wheel can be set to a constant speed. The one-arm drive wheelchair developed for bowling competition, as shown in Figure 1b, has a structure incorporating no camber angle because it must specialize in straight-line driving based on the movement characteristics associated with competition. These two competitive wheelchairs, each of which can be driven with one arm, were manufactured by Ox Engineering Co., Ltd. (Funabashi, Japan).

Following is a description of straight-line operation of the wheelchair using one arm. The x and y shown as markers in Figure 1a represent the two hand-rims. Regarding the two-handed rims attached to the right-hand drive side, the outer hand-rim shown in “x” operates the opposite left-hand drive wheel. The inner hand-rim shown in “y” operates the right-hand-drive wheel. When the dual hand-rims are operated simultaneously, the driving force is transmitted to both drive wheels. Thereby, the vehicle can move straight ahead. The manual propulsion action necessitates that one arm and hand exert repetitive force to the dual hand-rims accordingly.

### 2.2. Participants

The research participants in these experiments gave informed consent to serve as a study subject in the experiment. This study and use of the experimentally obtained data were approved by the Ethics Committee of the Nagaoka University of Technology (H30-1, H30-2). Research participants in the first experiment were two healthy men (173.5 ± 1.5 cm height; 70.5 ± 2.5 kg weight). Research participants in the second experiment were seven healthy men (173.1 ± 4.2 cm height; 65.4 ± 5.6 kg weight). All research participants, for whom the right hand was dominant had experienced adequate training in wheelchair manipulation. We want to explain changes and differences in muscle activity in one arm and two arms for a competitive wheelchair with a dual hand-rim system. Accordingly, we recruited healthy athletes as research participants. We understand that the inclusion of able-bodied athletes is a limitation affecting the generalizability of the study results. Research participants with a disability might show clear differences in muscle activity between one-arm and two-arm propulsion. The research participants adjusted the footrest and seat of the wheelchair before starting the experiments. The seating was secured by placing a towel between the gap on either side of the seat surface.

### 2.3. Experiment Protocol

#### 2.3.1. First Experiment

**Figure 2:**
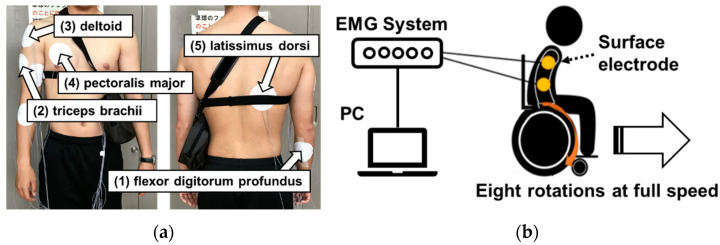
Measurement position of sEMG and schematic diagram I: (**a**) five measurement points and (**b**) schematic diagram of the experiment.

The purpose of this experiment is to use iEMG results to gain insight into differences in arm and shoulder joint muscle activation of a user during one-arm and two-arm use of our competitive wheelchair equipped with a dual hand-rim system. The driving force provided with the push rims is defined as the delivery of propulsion to the wheelchair. The wheelchair speed is related directly to the magnitude and frequency of the propulsive action. In fact, competitive wheelchair propulsion techniques are divisible into two phases: drive and recovery [35,36]. The most important factor affecting this propulsive force is the drive phase. Five measurement points are presented in Figure 2a: (1) flexor digitorum profundus (pinky side), (2) triceps brachii, (3) deltoid, (4) pectoralis major, and (5) latissimus dorsi. These muscles were selected for their well-known contribution to the drive and recovery phases. Figure 2b is a schematic diagram depicting the experiment. The procedure used for this experiment is the following.

**Figure 3:**
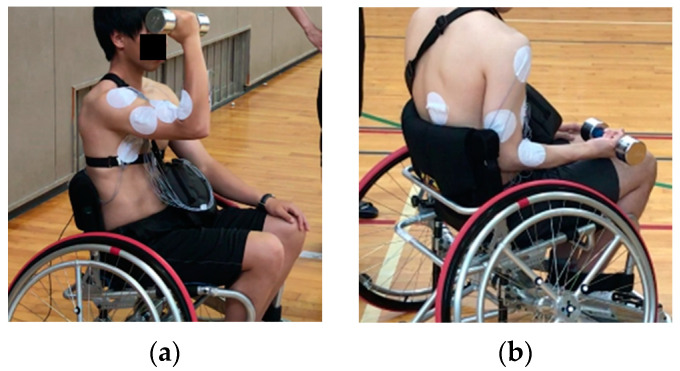
Posture of research participants under loading: (**a**) posture of research participants holding the dumbbells Part 1 and (**b**) posture of research participants holding the dumbbells Part 2.

1. As shown in Figure 3a,b, research participants hold a dumbbell with a load of 5 kg for 30 s before running. All subjects are loaded to produce identical muscle fatigue because it inhibits the possibility of inducing muscle fatigue bias through individual differences.
2. The start is made from a stationary position. During running, the hand-rim operation is performed once per second (for one rotation) for eight repetitions (for eight rotations) at full speed. The minimum number of cycles required for the wheelchair pro-pulsion to reach its maximum value from the stationary position is assumed because many competitions necessitate rapid acceleration in fewer cycles.
3. After completing running, the research participants take sufficient rest to recover from fatigue.
4. Steps 1–3 are performed alternately: three times in the case of one-handed running and three times in the case of two-handed running.

#### 2.3.2. Second Experiment

**Figure 4:**
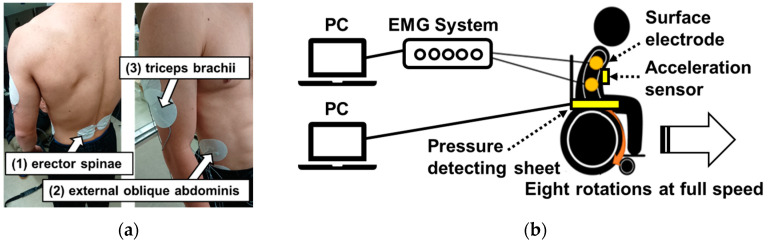
Measurement position of sEMG and schematic diagram II: (**a**) three measurement points and (**b**) schematic diagram showing the experiment.

The purpose of this experiment was to use iEMG data to estimate the movement posture during wheelchair propulsion with one-arm operation. Based on the hypothesis produced in light of the first experiment results, this experiment also measured the seat pressure distribution and three-dimensional movement of the upper limb synchronized with the sEMG findings. These results corroborate evidence obtained for body posture effects during one-arm propulsion of the wheelchair. The wheelchair used for this experiment had no camber angle, as shown in Figure 1b because the experiment specifically examines the operating posture during straight-line operation. Three measurement points are shown in Figure 4a: (1) erector-spinae, (2) external oblique abdominis, and (3) triceps brachii. Not all measurement points are shown in Figure 4a, but six measurement points were used for these surface EMGs because they were prepared for the left and right sides of the body. These muscles were selected for their well-known contributions to the drive phase [37]. A schematic diagram of the experiment is presented in Figure 4b. The procedures used for this experiment were the same as those described in 1–4 of Section 2.4.1.

### 2.4. Data Recording and Analysis

#### 2.4.1. Measuring Instruments

The following is a description of the measuring instruments used for data collection, which included a surface electromyogram (PolymatePro MP6000 biological signal system; Miyuki Giken Co., Ltd., Bunkyo, Japan), a pressure-detecting sheet (SR Soft Vision; Sumitomo Riko Co., Ltd., Nagoya, Japan), and a three-axis acceleration sensor (MyBeat; Union Tool Co., Tokyo, Japan). The sampling frequencies used for sEMG were 20–2000 Hz, with impedance of 250 GΩ and 24 input channels. For EMG data, surface electrodes were attached to the measurement position of the agonist muscle necessary for hand-rim operation. Details of its position are presented in the experiment protocol. The electrical signals obtained from the electrodes were recorded using a biological signal system (PolymatePro MP6000; Miyuki Giken Co., Ltd.) to a PC connected to the system at a sampling frequency of 1 kHz. The 450 × 450 mm pressure-detecting sheet included 256 pressure sensor elements. The sampling frequency was 5 Hz. The measurement range of pressure values was 0–200 mmHg. The right side of the lateral direction of the wheelchair was the X-axis positive direction. The front direction was the Y-axis positive direction. The three-axis acceleration sensor measured body axis movement. The right side of the lateral direction of the wheelchair was the X-axis positive direction. The vertically upward direction was the Y-axis positive direction. The front direction was the Z-axis positive direction. The sampling frequency was 128 Hz. The acceleration range was ±4 G.

#### 2.4.2. Data Analysis

Muscle activation, which is described as the linear envelope of the EMG signal [38], has been studied quantitatively using iEMG [39]. In an earlier study, sEMG findings of wheelchair users were used as indicators of wheelchair propulsion because a correlation exists between sEMG data and muscle strength [31]. For this study, sEMG measurements were taken at the arm, shoulder, and trunk muscles related to hand-rim operations. Re-search participants were prepared for placement of EMG electrodes at the measurement position by wiping the skin with alcohol and by lightly abrading it. Next, sufficient electrode paste (Ten20 Conductive; Weaver and Co., Aurora, CO, USA) was applied inside the surface Ag/AgCl EMG electrodes (MA-C001-15; Fukuda M-E Kogyo Co., Ltd., Nagareyama, Japan) to slightly overfit it. Then the electrode was placed onto the measurement position and pressed firmly. The electrodes were secured with surgical tape to minimize displacement during movement. A ground electrode was placed on a bony site over the iliac bone. For this study, sEMG data from each muscle were collected at a sampling frequency of 1 kHz during eight cycles for each research participant. One cycle defined here is one stroke of the hand-rim operation (to recovery phase from drive phase). The raw sEMG data were exported (BIMUTAS II; Kissei Comtec Co., Ltd., Matsumoto, Japan) for signal analysis and post-acquisition processing. A high-pass filter was used to remove noise. The integrated EMG (iEMG) was calculated using full-wave rectification smooth of sEMG data for each muscle for each research participant. In this study, the integrated value per second of iEMG measured at rest was normalized by the average value per stroke of iEMG measured during eight strokes of driving. These iEMG data quantitatively represent the total work-load of electrical activity of the muscles, meaning that the data quantify the amount of muscle activity for one drive during wheelchair operation.

The inclination of the body axis during wheelchair propulsion was measured using a three-axis acceleration sensor mounted in the center of the chest. The sampling frequency was 128 Hz. First, the sensor values in the three-axis (X-axis, Y-axis, Z-axis) were recorded when the research participants were held stationary in a competitive wheelchair for 10 s. We adopted the average of these values as our reference value at rest. Next, the difference between the results of all eight cycles and the reference value was then determined. Finally, we integrated the difference values for each cycle interval. The cycling interval is one second. However, individual differences occur. The samplings per cycle were adjusted by individually checking the peak values of the measured data. The result is presented as the inclination of the body axis during the wheelchair propulsion cycle. The reason for grouping them into eight cycles is to synchronize them with the iEMG results.

The seat pressure distribution during wheelchair propulsion was measured using a pressure-detecting sheet. The sampling frequency was 5 Hz. The sensor elements in a pressure-detecting sheet are arranged longitudinally and horizontally (16 × 16). The sheet was divided into nine grids because we wanted to classify the direction of the pressure distribution horizontally, vertically, and diagonally. The total sensor elements inherent in one grid were set to 32 (6 × 6). First, the sensor values in the seat pressure were recorded when the research participants were held stationary in a competitive wheelchair for 10 s. We adopted the average in the one grid as each reference value of static seat pressure. Next, the difference between the results of eight cycles in each grid and the reference values was then determined. Finally, we add the difference values for eight cycles. The result was recorded as the seat pressure distribution during the wheelchair propulsion cycle. These values are used as validation data for the body posture estimation.

#### 2.4.3. Statistical Analysis

Data were analyzed using software (RStudio, ver. 1.4.1106: GNU Affero General Public License). For the first experiment, iEMG data from five muscles were analyzed: flex-or digitorum profundus (pinky side), triceps brachii, deltoid, pectoralis major, and latissimus dorsi. For the second experiment, iEMG data from six muscles were analyzed: erector spinae (right and left side), external oblique abdominis (right and left side), and triceps brachii (right and left side). Shapiro–Wilk’s test revealed normality of the iEMG data in each experiment [40]. To assess significance of differences, a Wilcoxon signed-rank test by non-parametric data was selected for the corresponding two groups (one arm and two arms) [41]. The Friedman test was selected for the four groups of the corresponding non-parametric data. Furthermore, differences in means between the two groups in the four groups of data were selected with the Tukey honestly significant difference test. All thresholds for significance were set at the *p* < 0.05 level of confidence. The effect size was based on Cohen’s report [42].

## 3. Results

### 3.1. Results of iEMG for Muscle Activation during One-Arm Propulsion

**Figure 5:**
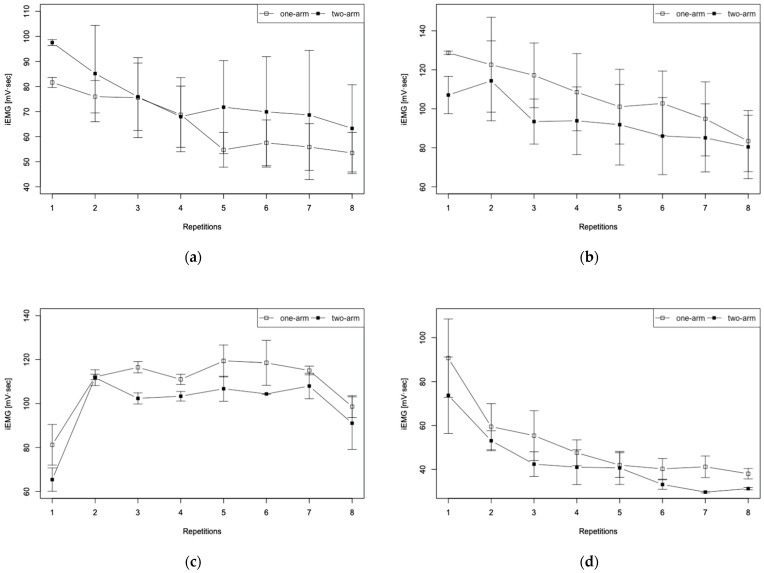

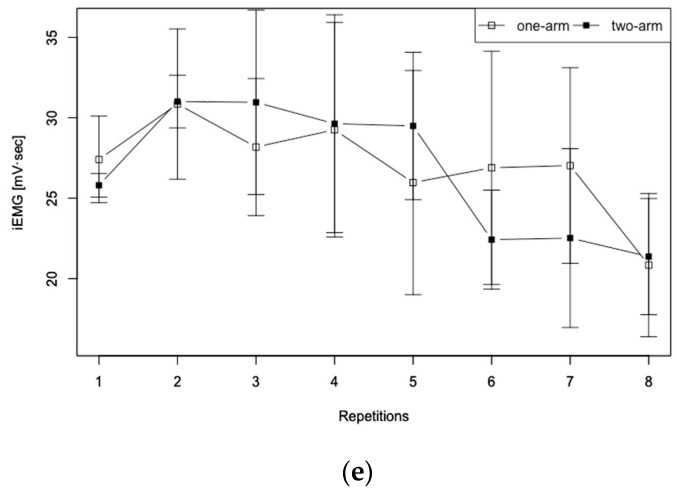
iEMG calculation results for eight strokes performed with one-arm and two-arm driving: (**a**) flexor digitorum profundus (pinky side), (**b**) triceps brachii, (**c**) deltoid, (**d**) pectoralis major, and (**e**) latissimus dorsi.

The graph portrayed in Figure 5 depicts the transition per stroke for one-arm and two-arm driving up to eight strokes. The average of iEMG data obtained for all trials is shown. The results are iEMG data for (a) flexor digitorum profundus (pinky side), (b) tri-ceps brachii, (c) deltoid, (d) pectoralis major, and (e) latissimus dorsi. Error bars in the figure represent the standard error.

**Figure 6:**
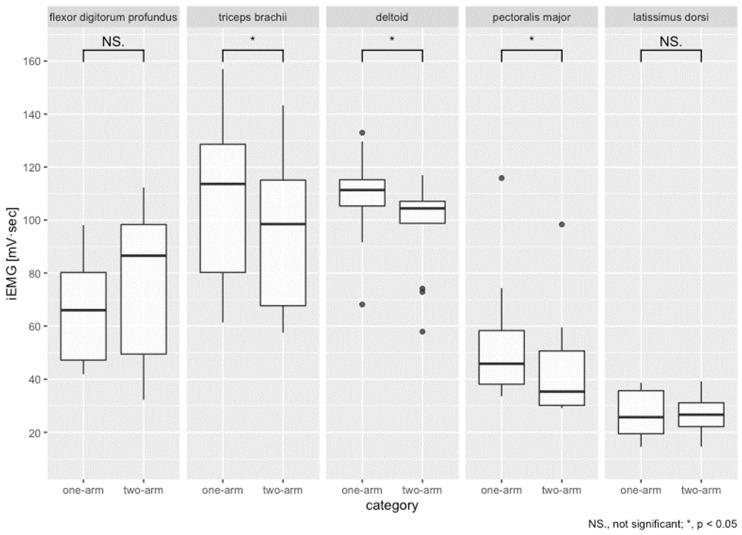
Distribution of iEMG results obtained at each muscle site with one-arm and two-arm driving.

As shown in Figure 6, a Wilcoxon signed-rank test using non-parametric data found significant differences in muscle activation between one-arm and two-arm propulsion were found for the triceps brachii (*p* = 0.0000, *r* = 1.04), deltoid (*p* = 0.0013, *r* = 0.80), and pectoralis major (*p* = 0.0002, *r* = 0.95), with one-arm data indicating greater muscle activation for the three previously described muscles compared to propulsion using two arms. The flexor digitorum profundus deltoid (*p* = 0.1167, *r* = 0.39) and latissimus dorsi (*p* = 0.8603, *r* = 0.04) muscle activation iEMG data were not significantly different between data obtained for one arm and for two arms.

### 3.2. Results of iEMG for Body Posture Estimated during One-Arm Propulsion

#### 3.2.1. Differences in Muscle Activation Because of Different Driving Patterns

**Figure 7:**
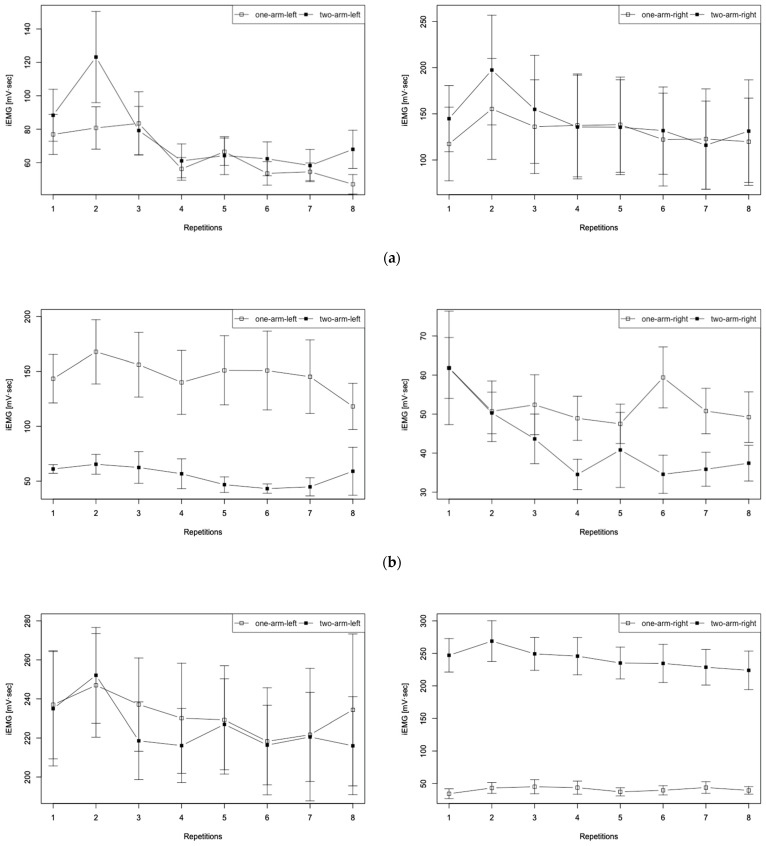
iEMG calculation results for eight strokes during one-arm and two-arm driving: (**a**) erector spinae, (**b**) external oblique abdominis, and (**c**) triceps brachii.

**Figure 8:**
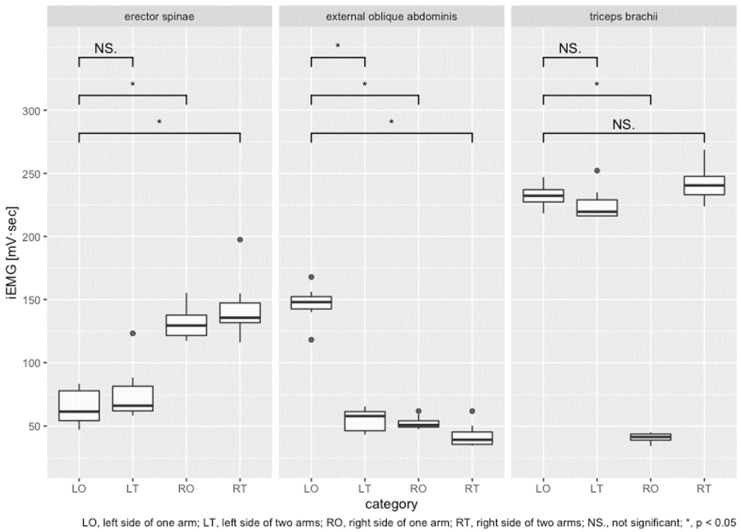
Distribution of iEMG results obtained at each muscle site with one-arm and two-arm driving (by left and right side).

The iEMG results obtained for the erector spinae, external oblique abdominis, and the triceps brachii of the research participants’ collaborators in the experiment are presented in Figure 7. The graph presents iEMG findings obtained up to a total of eight strokes with one-arm and two-arm driving, divided into those for the left-side measurement site (a) and for the right-side measurement site (b), centered on the body axis. It is noteworthy that the iEMG data at each stroke are average values of all trials. The error bars in the figure represent the standard error. Results for the distribution of iEMG data at each muscle site are presented in Figure 8 with a box plot. As shown in Figure 8, nonparametric tests were performed using the Friedman test (*p* < 0.05) for four groups of target muscle sites: left side of one arm, left side of two arms, right side of one arm, and right side of two arms. Results showed differences in the representative values among the four groups: erector spinae was *p* = 0.0002, external oblique abdominis was *p* = 0.0002, and triceps brachii was *p* = 0.0001.

Next, Table 1 presents test results for the difference in means between the two groups for the four groups of data. The Tukey honestly significant difference test was applied. The results obtained for one-arm operation showed a significant difference in muscle activity between the left and right. The results also confirmed that one-arm and two-arm operations produced differences in the erector spinae and external oblique abdominis.

#### 3.2.2. Rate the Body Axis and Seat Pressure Distribution

**Figure 9:**
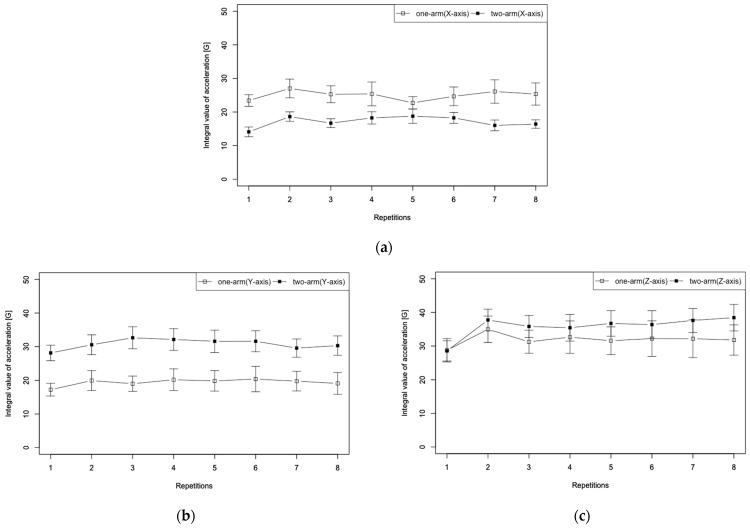
Body axis change results obtained for one-arm and two-arm driving for eight repetitions: (**a**) X-axis, (**b**) Y-axis, and (**c**) Z-axis.

Results obtained during one-arm and two-arm operation and then presented in Figure 9 show changes in the body axis from the three-dimensional acceleration sensor attached to a user’s chest for eight cycles. Figure 9a presents results obtained for the lateral direction of the body (X-axis), (b) results obtained for the vertical direction of the body (Y-axis), and (c) results obtained for the front–back direction of the body (Z-axis). Error bars in the figure represent the standard error. Wilcoxon signed-rank test results obtained from non-parametric data indicated significant differences in muscle activation found for the X-axis (*p* = 0.0078, *r* = 0.94), Y-axis (*p* = 0.0078, *r* = 0.94), and Z-axis (*p* = 0.0156, *r* = 0.85).

**Figure 10:**
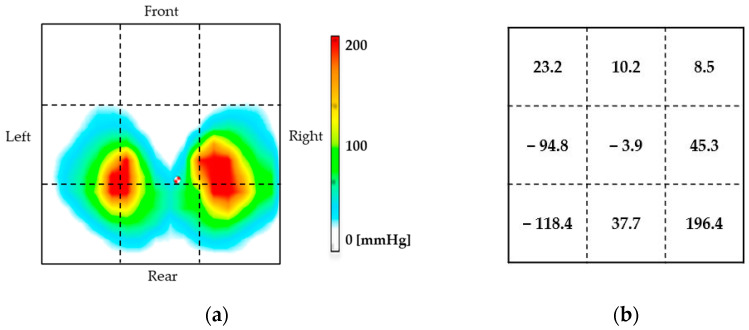
Different seat pressure distribution values for the nine segments: (**a**) an example of a heat map at rest, (**b**) the difference results for seat pressure distribution.

Results shown in Figure 10 were obtained by dividing 256 seat pressure distribution values with measurement resolution of 16 × 16 into nine sections, then calculating the difference from the seat pressure reference value for each experiment. Thereafter, the results of one arm to two-arm operation were subtracted from the total pressure values for each divided area. The reason for dividing the data into nine parts was to elucidate the direction of the user’s center of gravity movement. Figure 10a presents an example of a heat map visualization of the seat pressure distribution at rest, which is the reference value for seat pressure. Figure 10b shows the results of differencing the seat pressure distribution values from one-arm to two-arm operation.

## 4. Discussion

### 4.1. Muscle Activation Evaluation Using iEMG during One-Arm Propulsion

For EMG data recorded during the first experiment, the EMG data for one stroke of the hand-rim operation were extracted. After full-wave rectification, the iEMG data were calculated. These iEMG data quantitatively represent the total workload of the electrical activity of the muscles, meaning that they represent the amount of muscle activity for one drive during wheelchair operation. The graph presented in Figure 5 depicts the transition per stroke for one-arm and two-arm driving up to eight strokes. It is apparent from this figure that the (a) flexor digitorum profundus, (b) triceps brachii, and (d) pectoralis major, showed a large increase in muscle activity at the first stroke and a monotonic decrease with each increase in the number of strokes. For one-arm driving as well, these muscles indicate that the greatest amount of muscle force is necessary at the start of driving. Subsequently, the load on these muscles during the push phase decreased concomitantly with increasing wheelchair speed. Consequently, muscle activation also decreased concomitantly with the increasing stroke rate. In fact, the (c) deltoid and (e) latissimus dorsi results indicate that the amount of muscle activity decreased at the first stroke and end strokes for both one-handed and two-handed driving. Results obtained for the (c) deltoid showed marked changes in muscle activity from the first stroke to the second stroke. Moreover, results found for the (e) latissimus dorsi showed a gradual decrease in muscle activity from the second stroke. These results also indicated a similar trend for both one-handed and two-handed driving, suggesting that these muscles are the main driving muscles playing a fundamentally important role in speed maintenance or acceleration movements even in one-handed driving.

Next, we use statistical analyses for specific examination of muscle sites that are significantly different. In the (b) triceps brachii during one-arm operation, the muscle activation increased by about 14% compared to that during operation with two arms. In the (c) deltoid and (d) pectoralis major during one-arm operation, muscle activation increased by about 10% and 20% compared to that during operation by two arms. These muscle sites were found to play an important role during one-arm operation. The reason for this large role is that, when one correlates the results of the decrease in muscle activity from two to seven strokes with the increase in muscle activation of about 10% over one arm, it can be shown to have the strongest effect on speed maintenance and acceleration movements among the driving muscles that were measured. However, the amount of these muscle activations during one-handed driving was less than expected because the competitive wheelchair used for the experiment requires only one arm to transmit the propulsive force to the opposite drive wheels. A simple calculation indicates that one-arm operation requires twice as much muscle activation. In conclusion, the experimentally obtained results suggest that only one arm and other factors can maintain muscle activation producing the same propulsive force as that produced by two arms.

### 4.2. Body Posture Estimation Using iEMG for Evaluation during One-Arm Propulsion

#### 4.2.1. Differences in Muscle Activation Attributable to Different Driving Patterns

The graph shown in Figure 7 presents iEMG findings up to eight strokes with one-arm and two-arm driving, divided into the left-side measurement site (a) and the right-side measurement site (b), centered on the body axis. The iEMG data of the erector spinae in the upper part of Figure 7 show similar trends for (a) the left side and (b) the right side. However, the trends of the external oblique abdominis shown in the middle part and the triceps brachii presented in the lower part are very different between the left and right sides. For the external oblique abdominis, a constant difference was found in the transition of each stroke between one arm and two arms on the left side. For the triceps brachii, a constant difference was found on the right side. The results confirmed that one-arm and two-arm operations produced differences in the external oblique abdominis and triceps brachii.

Next, we specifically examine categories for which significant differences were found based on one-arm operation according to the results of the statistical analyses for muscle activation. Muscle activation in the erector spinae was approximately 49% less on the left side (LO) than on the right side (RO) of the hand-rim operation. The left side (LO) was about 1.5 times more than the right side of two arms (RT). On the opposite side (RO) of the hand-rim operation, the number was approximately 1.7 times more than on the left side with two arms (LT). The erector spinae, long, large muscles located in the back, were found to be activated on the opposite side of the hand-rim operation. The erector spinae muscles are mainly involved in trunk extension movements. The role of the erector spinae is to extend (retroflex) the trunk during athletic movements. This role contributes to posture maintenance, stabilizing the upper body in all sports activities. The erector spinae are also involved in upper-body raising activities. The correct operating posture for straight running in two-arm operation is that the body axis does not tilt in the left–right direction. In addition, the upper limbs bend forward and backward repeatedly. Muscle activation of the erector spinae, which constitute the trunk of the user, is probably not biased to either side to any considerable degree. Therefore, one-arm operation during straight running might cause the user’s movement body posture to be biased not only to forward bending and backward bending but also to the left or right, considering the effects of the decrease in muscle activation.

In the external oblique abdominis, left side (LO) muscle activation was about 2.7 times greater by than at other times. The external oblique abdominis were found to play an important role in the wheelchair propulsion force by one-arm operation. The external oblique abdominal muscles are the most superficial muscles on the lateral side of the abdomen. They are used for trunk rotation, and are also involved in other functions such as bending the trunk sideways. The role of the muscle is mainly to rotate, flex (forward bending), and bend the trunk laterally during locomotion. The muscles therefore contribute greatly to all motion behavior that twists the body. As described above, the bimanual drive-in straight-line running repeats only forward and backward bending of the upper limbs. Results indicate that the muscle activity of the left external oblique abdominis, which operates the hand-rim, increased nearly threefold during one-arm operation. The user’s operating body posture includes lateral flexion and rotation of the upper limb in addition to flexion. This prediction can be clarified from results of the three-axis acceleration sensor attached to the upper limb and the change in the seat pressure distribution described below.

In the triceps brachii, right side (RO) muscle activation decreased 80% compared to that during the left side (LO). This is a reasonable result because the right arm, which does not operate the hand-rims, is placed on the user’s lap; it is not moved. The triceps brachii extends the elbow joint. It is the main muscle used for the push phase of wheelchair pro-pulsion. The long head of the triceps, a so-called biarticular muscle, is also involved in shoulder joint internal rotation and extension. The muscle role is to throw, push, and lift objects during athletic activities. It contributes greatly to sports activities that involve pushing forward. As shown by the first experiment, one-arm operation exhibited the highest muscle activity near the beginning of the drive, which increased by more than 10% compared to two-handed driving. However, comparisons between one arm (LO) and two arms (LT) revealed no significant differences in muscle activation.

#### 4.2.2. Evaluation of Posture Estimation

This section presents discussion of the validity of posture estimation using iEMG based on the user’s body axis and the amount of change in the seat pressure distribution. As shown in Figure 9, no characteristic changes were observed between cycle segments for the three axes. These results suggest that both one-arm and two-arm operations were cyclically repeated stably. In addition, the stable body posture during wheelchair propulsion suggests that we have also reduced the effect of postural changes on muscle activation. The amount of change in the transverse direction of the body axis (X-axis) increased approximately 1.5 times when averaged over all cycles during one-arm operation. However, the vertical direction of the body (Y-axis) increased by about 1.6 times during two-arm operation. The front–back direction (Z-axis) also increased by about 1.1 times in two arms. Additionally, it can be confirmed that the amount of change in the forward–backward direction is greatest for both one-arm and two-arm operations. The one-arm operation causes a forward tilt of the driving side (left side). The two-arm operation would tilt toward the direction of travel. The increase in the X-axis during one-arm operation suggests the influence of flexion and extension of the external oblique abdominis due to the trunk circumnutating motion. The explanation in Section 4.2.1 corroborates the results.

Figure 10b shows that the seat pressure distribution during one-arm operation showed an increase in pressure values on the opposite side (right side) of one-arm operation (left side) compared to two-arm operation. The maximum value of the difference was 196.4 (mmHg) at the rear of the right side. In the case of one-arm operation, the center of gravity of the seat pressure shifted to the opposite side of the hand-rim operation side to that found in the case of two arms. This result derives from movement of the center of gravity to the opposite side (right side) of the hand-rim when operating the hand rim with one arm, which reflects the effort to keep the body axis of the body centered in the operating body posture. To support this result, we have already explained, in Section 4.2.1, the muscle activation of the external oblique abdominis by one-arm operation, which are involved in torso rotation, flexion, and lateral bending, and increased muscle activation. Results also confirmed that the body position of the center of gravity on the seat surface moved to the right and rearward during movement to return the body axis to the center because of the influence of the increase in the amount of change in the body axis in the left oblique forward direction. In conclusion, we found that the body posture of one-arm operation is different from that of two-arm due to the effect of the activation site. These results suggest that an approximate motion posture can be estimated from observation of muscle activation by surface EMG.

## 5. Conclusions

This study was conducted to develop competitive wheelchairs that will allow participating in sports by facilitating straight-ahead movement with one arm. This wheelchair has the potential to be a new assistive technology for people participating in sports. However, the differences in muscle activation and body posture between one-arm and two-arm operations have not been clarified. Once these issues are resolved, it will be possible to determine the muscles needed to strengthen one-arm operation and the approximate range of adaptability to the site and degree of disability.

The first experiment, using iEMG results, was conducted to elucidate differences in muscle activation of a user’s arm and shoulder joint muscles during propulsion using only one arm using two arms with a competitive wheelchair equipped with our dual hand-rim system. One-arm operation showed significant differences in activation of the triceps brachii and deltoid and pectoralis major, with a 10–20% increase in muscle activation compared to that when using two arms. Nevertheless, the amounts of muscle activation during one-handed driving were less than expected. The experimentally obtained results suggest that only one arm and other factors can provide the same propulsive force as that supplied by two-arm driving.

The second experiment was undertaken to use iEMG data to estimate the movement posture during wheelchair propulsion with one-arm operation. For the erector spinae, muscle activation was approximately half on the hand-rim manipulation side (left) com-pared to the right side. For the external oblique abdominis, the left-side muscle activation was about 2.7 times greater than at other times. For the triceps brachii, comparisons be-tween the left side of one arm and the left side of two arms during hand-rim manipulation revealed no significant difference in muscle activation. The external oblique abdominis muscles played an important role in producing wheelchair propulsion during one-arm operation. Finally, the validity of using iEMG for posture estimation was verified by the amount of change found in the user’s body axis and seat pressure distribution. In conclusion, results indicate that one-arm wheelchair operation activates different muscle sites and produces different body posture than wheelchair manipulation by two arms, thereby allowing movement and posture estimation using surface EMG.

## Figures and Tables

**Table 1:** Tukey honestly significant difference test.

Pairing	*p*-Value (*p* < 0.05)		
	Erector Spinae	External Oblique Abdominis	Triceps Brachii
LO–LT	0.6764	0.0000	0.6117
LO–RO	0.0000	0.0000	0.0000
LO–RT	0.0000	0.0000	0.2851
LT–RO	0.0000	0.9662	0.0000
LT–RT	0.0000	0.0799	0.0239
RO–RT	0.5735	0.1956	0.0000

LO, left side of one arm; LT, left side of two arms; RO, right side of one arm; and RT, right side of two arms.
